# Waste Sunflower Oil as a Feedstock for Efficient Single-Cell Oil and Biomass Production by *Yarrowia lipolytica*

**DOI:** 10.3390/foods15020290

**Published:** 2026-01-13

**Authors:** Bilge Sayın

**Affiliations:** Department of Gastronomy and Culinary Arts, School of Tourism and Hotel Management, Ardahan University, Ardahan 75002, Türkiye; bilgesayin@ardahan.edu.tr

**Keywords:** single-cell oil, Taguchi design, bioconversion, sustainable valorization, renewable feedstocks, palmitic acid

## Abstract

In this study, single-cell oil (SCO) production from waste sunflower oil was optimized using *Yarrowia lipolytica* IFP29 (ATCC 20460). Optimizations were performed via a multi-response approach based on the Taguchi orthogonal array design (L16), targeting maximum biomass concentration and lipid content (based on dry cell weight). A total of 16 experimental conditions were tested with five key parameters: nitrogen concentration (0, 1, 2, and 4 g/L), WCO concentration (20, 40, 60, and 80 g/L), Tween 80 content (0, 0.5, 1, and 2%) as well as the application of sonication and sterilization. Analysis of variance revealed that all tested factors, except Tween 80 and sonication, had statistically significant effects on lipid content (*p* < 0.05). The highest lipid content (72.86% of dry cell weight) was obtained in a sterilized, sonicated medium containing 80 g/L WCO and 2% Tween 80, under conditions without nitrogen supplementation. In contrast, maximum biomass production (4.18 g/L) was achieved in sterile cultures with high nitrogen (4%) and high WCO (80 g/L) in the absence of Tween 80 and sonication. Palmitic acid (C16:0) content was also successfully optimized, with nitrogen concentration and Tween 80 supplementation exerting a statistically significant effect (*p* < 0.05). These results highlight the potential of waste sunflower oil as a low-cost feedstock for SCO production and support the development of economically and environmentally sustainable bioprocesses.

## 1. Introduction

The global generation of waste vegetable oils is estimated to exceed 15 million tons annually, of which approximately 1 million tons are produced each year within the European Union [1]. This substantial volume is closely linked to the extensive and increasing global consumption of edible oils. In 2023/2024, worldwide vegetable oil consumption reached 218.15 million MTs, with sunflower oil accounting for 20.65 million MTs and ranking fourth, following palm, soybean, and rapeseed oils [2]. Sunflower oil, obtained from *Helianthus annuus* seeds, is characterized by a high linoleic acid content (45–74%), very low linolenic acid levels (<0.3%), moderate oleic acid (14–43%), and relatively low saturated fatty acids (8–17%) [3]. Although palm and soybean oils dominate global vegetable oil production, sunflower oil constitutes a notable market share (9.7%). Due to its widespread use, particularly in frying applications, sunflower oil is expected to contribute substantially to the overall generation of waste cooking oils (WCOs) [4].

WCOs comprise various waste oil streams, including oils generated from deep-frying operations, reclaimed oils from catering services, and so-called gutter oil. When improperly managed, such oils may compromise consumer rights and food safety, thereby posing direct risks to public health. These risks arise because WCO often contains hazardous compounds such as heavy metals, aflatoxins, and benzopyrene [5]. WCO may also contain harmful oxidation or microbial by-products and is associated with risks such as gastrointestinal disorders and cancer [6]. Furthermore, the disposal of WCO has become a pressing global issue, as it is commonly classified as domestic waste and generated in large volumes by fast-food outlets, hotels, and the food industry [7]. When released into wastewater streams, WCO can interact with other constituents to form fat, oil, and grease deposits, leading to severe pipeline blockages. These deposits also release pollutants into the environment and expose living organisms to pathogenic risks [8]. Additionally, improper disposal has been linked to oxygen depletion in aquatic systems and unpleasant odors in the atmosphere [9]. Despite these risks, WCO remains an attractive feedstock owing to its low cost, widespread availability, and high recycling potential [10].

The term single-cell oils (SCOs) was introduced to distinguish microbial lipids produced by single-celled organisms, considered suitable for human use, from conventional plant- and animal-derived oils. While SCOs are universal components of all microorganisms and generally constitute approximately 6–8% of dry cell weight, microorganisms that accumulate more than 20% (*w*/*w*) of their dry biomass as lipids are classified as oleaginous [11]. Several microorganisms, including *Yarrowia lipolytica*, *Apiotrichum curvatum*, *Mortierella isabelina*, *Schizochytrium limacinum*, and *Rhodosporidium toruloides*, are notable for their ability to synthesize and accumulate large quantities of unsaturated fatty acids. Consequently, these species have become a major focus of extensive research [12]. Among oleaginous yeasts, *Y. lipolytica* serves as the principal model species owing to its well-characterized lipid metabolism, extensive genetic toolbox, and fully sequenced genome [13]. Accordingly, *Y. lipolytica* exhibits lipid accumulation that is strongly influenced by several factors, including nitrogen and phosphorus sources, pH, temperature, and the carbon-to-nitrogen (C/N) ratio [14].

SCOs, rich in polyunsaturated fatty acids (PUFAs), are valuable for nutritional enhancement and have been explored as sustainable alternatives or partial substitutes for conventional vegetable fats, such as palm oil and cocoa butter [15]. Over the past decades, they have gained considerable attention due to their broad applicability in supplementary diets and infant formulations, as well as in biodiesel production and the development of high-value products, including nutraceuticals, antimicrobial agents, and cosmetic ingredients [12]. Compared with plant- and animal-derived oils, microbial oil production offers faster growth rates, greater flexibility in substrate utilization, and independence from geographic and climatic constraints. Moreover, oleaginous microorganisms can be genetically engineered to enhance lipid yield and tailor fatty acid composition, and the lipids they accumulate closely resemble plant oils in terms of fatty acid and triacylglycerol profiles, supporting their suitability for nutraceutical applications [16]. Also, SCOs have emerged as a promising third-generation feedstock for biodiesel production, owing to their renewable nature, reduced CO_2_ emissions, and high lipid productivity [17].

Meanwhile, the continuous growth of the global population has increased the demand for food, feed, fuels, and other fossil-based products, resulting in higher per capita waste generation and greater environmental pressures. These challenges underscore the urgent need to move beyond the conventional linear production paradigm of “take, make, and dispose”. In this context, the circular economy framework has been proposed to enhance resource efficiency and reduce the economic, environmental, and social burdens associated with linear systems. This model offers several advantages, including waste valorization, reduced ecological footprints, and the sustainable production of bioenergy and biochemicals [18]. On the other hand, the economic feasibility of biotechnological processes largely depends on the availability of low-cost substrates for microbial cultivation, as nutrients alone may account for up to 70% of the total production cost. For this reason, residual materials from forestry, agriculture, and the food industry have been widely investigated as alternative feedstocks for SCO production [19]. Among these, WCOs have emerged as a particularly promising option, offering both economic benefits and a sustainable solution to a critical environmental challenge.

Valorization of WCO through biotechnological processes, particularly using oleaginous yeasts, represents a sustainable strategy to convert processed waste into valuable bioproducts. In this process, triacylglycerols, the main constituents of WCO, are hydrolyzed by lipases and subsequently assimilated by yeast cells for lipid biosynthesis [20]. Accordingly, the primary objective of this study is to optimize SCO production using WCO as a low-cost and sustainable carbon source. To this end, *Y. lipolytica* was employed, and key process variables (nitrogen concentration, substrate level, Tween 80 supplementation, sonication, and sterilization) were evaluated using the Taguchi method to simultaneously maximize biomass formation and lipid accumulation. The novelty of this work lies in demonstrating that process optimization enables the controlled modulation of fatty acid composition, particularly palmitic acid, in the resulting SCOs.

## 2. Materials and Methods

### 2.1. Microorganism and Culture Conditions

*Y. lipolytica* IFP29 (ATCC 20460) was obtained from the American Type Culture Collection (ATCC, Manassas, VA, USA). The strain was cryo-preserved at −80 °C in Malt Extract Broth (MEB) containing 50% (*v*/*v*) glycerol and reactivated in MEB before use.

### 2.2. WCO Collection and Characterization

In this study, a single, well-defined WCO was deliberately selected to minimize substrate variability and enable a controlled evaluation of the substrate–microorganism relationship. Given the inherent heterogeneity of WCOs and the fact that small and medium-sized enterprises typically do not use pure sunflower oil or exclusively fry potatoes, a standardized frying protocol was implemented to ensure consistency and reproducibility. WCO was produced by frying potatoes using a commercially available sunflower oil in a household-type deep fryer. The frying process was conducted at 180 °C and terminated once the oil’s polarity reached 16, as measured using a Testo 270 frying oil tester (Testo, Lenzkirch, Germany). The fatty acid composition and Fourier Transform Infrared Spectroscopy (FTIR) spectra of the obtained oil sample, as well as the rationale for selecting *Y. lipolytica* IFP29 for this study, are detailed in the work of Sayın et al. [21].

### 2.3. Lipid Production Medium and Fermentation Conditions

Lipid production was performed in a medium containing (g/L): Na_2_HPO_4_ 2.5, CaCl_2_·2H_2_O 0.15, MnSO_4_·H_2_O 0.04, ZnSO_4_·7H_2_O 0.02, FeCl_3_·6H_2_O 0.15, and MgSO_4_·7H_2_O 1.5. (NH_4_)_2_SO_4_ was used as the sole external nitrogen source, and media without (NH_4_)_2_SO_4_ were considered nitrogen-limited due to the absence of added nitrogen and were employed to promote lipid accumulation. 

Prior to inoculation, WCO and Tween 80 were premixed by vortex homogenization to promote uniform emulsification. In experimental runs where sonication was applied, the WCO-containing medium was additionally subjected to low-energy sonication using an ultrasonic water bath (40 kHz, 1 min) in order to enhance the dispersion of hydrophobic substrates. Furthermore, sonication applied during lipid extraction (Section 2.5) served a different purpose and was performed under distinct conditions. This sonication step was performed at low intensity and short duration and did not induce cell disruption or lipid release.

The medium was sterilized at 121 °C for 15 min in an autoclave. Fermentations were conducted in 250 mL Erlenmeyer flasks containing 50 mL of medium, inoculated with 1 mL of an exponentially growing pre-culture containing 10^6^ cells/mL. Cultures were incubated in a rotary shaker (JSSI-100; JS Research, Gongju, Republic of Korea) at 28 °C and 180 rpm for 6 days. The initial pH of the medium was adjusted to 6.5 and was not controlled during fermentation.

### 2.4. Determination of Biomass Concentration

To quantify biomass production, dry cell weight was measured. Culture broth was centrifuged at 5000 rpm for 10 min (Thermo Fisher MR23I, Waltham, MA, USA). Cell pellets were washed twice with distilled water to remove any remaining medium components without causing lipid loss. They were then dried at 95 °C until a constant weight was achieved and subsequently weighed [22].

### 2.5. Lipid Extraction and Quantification

Total lipid content was determined using a modified method incorporating intensive mechanical and ultrasonic disruption. Harvested biomass was treated with 8 mL of 4 M HCl and 0.7 mm diameter glass beads, followed by sonication in an ultrasonic water bath at 60 °C for 2 h, conditions specifically selected to ensure effective cell wall rupture and complete lipid release. Unlike the mild sonication applied prior to fermentation (Section 2.3), this extraction-stage sonication involved prolonged exposure and higher effective energy transfer and was applied uniformly to all samples. This ensured that lipid recovery efficiency was consistent across treatments and did not confound comparative lipid content analysis. The acid-hydrolyzed mixture was extracted with 16 mL of a chloroform/methanol (1:1, *v*/*v*) solution and stirred for 2–3 h at room temperature. Following centrifugation at 5000 rpm for 5 min, the organic (lower) phase was collected, and the solvents were evaporated at 40 °C under reduced pressure using a rotary evaporator (Buchi B-490, Flawil, Switzerland) [23]. Lipid content was expressed as the percentage of lipid based on dry biomass weight [24].

### 2.6. Preparation of Fatty Acid Methyl Esters (FAMEs)

For fatty acid composition analysis, 1.5 mL of 2 M methanolic NaOH was added to the extracted lipids, followed by nitrogen flushing and incubation at 80 °C for 1 h. After cooling, 2 mL of boron trifluoride-methanol complex was added, and the mixture was again incubated at 80 °C for 30 min under a nitrogen atmosphere. Upon cooling to room temperature, sequential additions of 1 mL hexane, 1 mL distilled water, and another 1 mL hexane were performed with thorough mixing after each step. The samples were then centrifuged at 5000 rpm for 5 min at 4 °C. The upper organic layer was transferred to tubes containing anhydrous sodium sulfate for drying and subsequently placed into gas chromatography vials after a final nitrogen flush, following the method of Metcalfe and Schmitz [25].

### 2.7. Gas Chromatography Analysis of FAMEs

Fatty acid methyl esters were analyzed using a gas chromatograph equipped with a flame ionization detector (GC-FID; Agilent 7820A, Santa Clara, CA, USA). Nitrogen was used as the carrier gas at a flow rate of 1 mL/min, along with hydrogen and dry air. Separation was achieved using a CP-SIL 88 capillary column (100 m × 250 μm × 0.20 μm; Agilent). The oven temperature program was set as follows: initial temperature of 100 °C held for 3 min; ramped at 3 °C/min to 200 °C and held for 5 min; then ramped at 4 °C/min to 250 °C and held for 10 min. Standard mixtures of FAMEs (FAME mix, 4-7801, Supelco, Bellefonte, PA, USA) were used for peak identification. Results were reported as relative percentage composition of total identified fatty acids.

### 2.8. Statistical Analysis

In this study, experimental optimization was performed using the Taguchi design approach, which is well-suited for systematically investigating the effects of multiple variables with a reduced number of experimental trials. The Taguchi method emphasizes improving process performance by minimizing variability rather than merely achieving a desired target. This is achieved by employing orthogonal arrays that enable efficient experimentation and robust design. A key analytical tool within this methodology is the Signal-to-Noise (S/N) ratio, which assesses the relationship between the desired output (signal) and the variation (noise) inherent to the process. By maximizing the S/N ratio, the influence of uncontrollable factors can be minimized, resulting in improved consistency and reliability of results. For this purpose, a mixed-level orthogonal array design [L16(4^3^ × 2^2^)] was employed to evaluate the influence of selected factors and their levels on biomass concentration and lipid content (Table 1). Experimental runs were conducted in duplicate according to the predefined design matrix. Statistical analysis was carried out using analysis of variance (ANOVA) to determine the significance of the main effects and their interactions on the responses. All experimental design steps and statistical evaluations were executed using Minitab software (version 19; State College, PA, USA). Results were expressed as mean values ± standard deviation.

## 3. Results and Discussion

### 3.1. Optimization of Biomass Production and Lipid Content

The Taguchi design results demonstrated that biomass formation and lipid accumulation exhibited distinct trends depending on the experimental conditions (Table 2). The highest biomass concentration was obtained in Run 16 (4.18 g/L; S/N: 12.42) under conditions of high nitrogen availability (4%) and high WCO concentration (80 g/L). Nitrogen is a key structural component of purine and pyrimidine bases in nucleic acids (DNA and RNA) and is essential for genetic inheritance, cellular proliferation, and protein synthesis [26]. In this context, the results indicate that high nitrogen availability promotes rapid cell growth and biomass accumulation. In contrast, the highest lipid content was achieved in Run 4 (72.86%; S/N: 37.25) under conditions without nitrogen supplementation, together with a high WCO concentration (80 g/L) and Tween 80 supplementation (2%). In addition to these conditions, the combined application of sonication and sterilization likely improved substrate accessibility, thereby facilitating lipid biosynthesis. While moderate WCO concentrations (40–60 g/L) supported lipid accumulation, very high WCO levels (80 g/L), particularly under nitrogen-sufficient conditions, tended to suppress lipid biosynthesis. A confirmation experiment conducted under the predicted optimal conditions yielded a biomass concentration of 3.99 g/L and a lipid content of 71.2%, confirming the reliability of the Taguchi model predictions.

Oleaginous microorganisms are known to accumulate lipids under nutrient-limited conditions in the presence of excess carbon. Among the various nutrients, nitrogen limitation represents the most effective and readily controllable trigger for lipid biosynthesis. Under nitrogen-limited conditions, reduced protein and nucleic acid synthesis constrains cell growth, whereas continued carbon assimilation is redirected toward lipid formation, resulting in enhanced triacylglycerol accumulation within intracellular lipid bodies [27]. High carbon-to-nitrogen (C:N) ratios (typically 50:1–150:1) are therefore commonly applied to induce nitrogen limitation. Consistent with previous reports, our results confirm that precise adjustment of nitrogen concentration in the fermentation medium is pivotal for balancing biomass formation and lipid productivity [28].

Gao et al. [29] developed a variable pH co-fermentation strategy using food waste and WCO for microbial lipid production. Mechanistic analysis revealed that fatty acid salts generated from WCO under alkaline conditions functioned as surfactants, improving lipid accumulation. Nevertheless, lipid production was inhibited at elevated WCO concentrations (29.2 g/L), which was attributed to restricted oxygen transfer arising from excessive hydrophobic substrate loading. In contrast, the present study demonstrated that *Y. lipolytica* sustained high lipid contents even at substantially higher WCO levels (up to 80 g/L), indicating that the inhibitory threshold of WCO is strongly strain- and process-dependent.

Surfactants are amphiphilic compounds containing both hydrophilic and hydrophobic moieties that preferentially accumulate at interfaces between phases of differing polarity and reduce surface and interfacial tension [30]. The incorporation of surface-active agents into fermentation media can influence microbial physiology by enhancing substrate availability, modifying membrane permeability, and promoting cellular growth and respiration [31]. Grubišić et al. [32] investigated the effects of Tween 80 on the growth and lipid biosynthesis of *T. oleaginosus* cultivated in a glucose-based medium with a C:N ratio of 207.4 mol/mol. Tween 80 concentrations ranging from 0 to 25 g/L (0–1.82% *v*/*v*) significantly enhanced lipid accumulation without affecting biomass formation, yielding a maximum lipid concentration of 6.86 g/L and a productivity of 2.29 g L^−1^ d^−1^ at 25 g/L, representing an increase of more than 60% compared to the control culture. In contrast to these findings, the addition of Tween 80 in the present study significantly influenced biomass formation but did not exert a statistically significant effect on lipid content. This suggests that while Tween 80 may improve substrate accessibility or membrane fluidity, it did not substantially redirect metabolic flux toward lipid biosynthesis under the tested conditions.

Sonication has been widely investigated as a process intensification technique for chemical and enzymatic reactions due to its ability to enhance substrate dispersion and mass transfer, thereby increasing reaction rates and yields [33]. In this study, a brief ultrasonic pre-treatment (1 min) was applied to the culture medium prior to incubation to evaluate its potential influence on SCO production by *Y. lipolytica*. However, sonication did not result in a statistically significant improvement in lipid content. This outcome may be attributed to the low intensity and short duration of the treatment, which were likely insufficient to alter the physiological state or membrane permeability of the yeast cells prior to fermentation. In general, sonication is frequently employed to intensify organic synthesis through ultrasound-induced cavitation, where bubble formation, growth, and collapse generate localized high-energy microenvironments within the liquid phase [33]. The short pre-treatment applied in this study may not have generated sufficient cavitational energy to influence lipid biosynthetic pathways in *Y. lipolytica*. By contrast, Jadhav et al. [34] developed an ultrasound-assisted, enzyme-catalyzed process for the synthesis of designer lipids from long-chain triglycerides and medium-chain fatty acids. Under optimized conditions, the ultrasound-assisted process achieved a maximum lipid yield of 92% (fatty acid: triglyceride ratio 4:1, 40 °C, 3% enzyme loading, 70% duty cycle, 240 W power, and 360 min reaction time), with the enzyme retaining catalytic activity over 10 reuse cycles. The resulting designer lipids exhibited high oxidative stability for up to 35 days, Newtonian flow behavior, and favorable visual properties.

Low-cost and readily available substrates, particularly agroforestry residues and organic wastes derived from food and agricultural systems, have attracted increasing attention for the production of industrially relevant fermentation products. Nevertheless, microbial contamination remains a major challenge during fermentation, often resulting in substantial economic losses, especially at the industrial scale. Traditionally, sterilization and strict aseptic operation have been regarded as essential measures to prevent contamination. In large-scale microbial lipid production facilities with an annual capacity of 10,000 tons, sterilization-related operations contribute measurably to process economics, accounting for approximately 1.4% of total utility costs and nearly 4% of the installed cost of fermentation infrastructure [35]. Consequently, non-sterile cultivation strategies are often favored because they reduce energy, labor, and time requirements and can be effectively implemented through the optimization of culture parameters that suppress undesirable microorganisms while supporting the target strain [36]. Hydrophobic substrates such as WCO may inherently restrict the growth of non-target microorganisms due to their poor biodegradability and limited utilization by common microbial contaminants. In light of these considerations, WCO was employed in this study as a carbon source to reduce overall process costs, and non-sterile operating conditions were implemented to enhance process efficiency.

Chitnis et al. [37] cultivated *Y. lipolytica* under non-sterile fed-batch conditions using a synthetic medium containing acetic acid as the sole carbon source. Contamination was monitored via flow cytometry and microscopy. Stationary-phase feeding improved biomass yield and productivity, with contamination remaining below 2% for up to 48 h, whereas maintaining a minimum acetic acid concentration enhanced lipid yield and productivity with similarly low contamination throughout the process. The maximum biomass and lipid yields reached 0.57 g biomass/g substrate and 0.17 g lipid/g substrate, respectively, with corresponding productivities of 0.085 g/L/h and 0.023 g/L/h. Santamauro et al. [38] demonstrated that *Metschnikowia pulcherrima*, previously regarded as non-oleaginous, can accumulate lipid contents of up to 40% when pH and temperature are properly adjusted. The strain’s acidophilic nature and inherent production of antimicrobial compounds further enabled its cultivation under low-cost, non-sterile conditions. In another study, Rakicka et al. [39] evaluated a lipid-engineered *Y. lipolytica* JMY4086 strain for SCO production in fed-batch cultures using molasses and crude glycerol under varying oxygenation and inoculum conditions. The highest lipid content (31% of cell dry weight) was obtained at low inoculum density, 800 rpm agitation, and 1.5 L/min aeration. Under nitrogen-limited chemostat operation (dilution rate 0.01 h^−1^; 250 g/L crude glycerol), the strain achieved a biomass concentration of 60 g cell dry weight/L and a lipid productivity of 0.43 g/L/h.

In Table 3, the results of the ANOVA based on the Taguchi experimental design for biomass production are presented. The analysis showed that Tween 80 was the most influential factor affecting biomass formation, contributing 51.18%, and was statistically significant (*p* < 0.05). The second most influential factor was the nitrogen concentration, with a contribution of 24.65% (*p* < 0.05). Additionally, WCO concentration accounted for 16.12% of the variation (*p* < 0.05). On the other hand, sonication (1.15%) showed only limited and statistically insignificant effects on biomass formation (*p* > 0.05). The low error value (1.73%) supports the reliability of the model, while the high R^2^ (98.27%), adjusted R^2^ (93.50%), and predicted R^2^ (72.26%) values indicate that the model explains the data to a large extent.

The ANOVA results (Table 4) clearly demonstrate that nitrogen concentration was the most influential factor for lipid content, contributing 52.55% of the total variance (*p* < 0.05). The second most significant factor was WCO concentration, with a contribution of 28.55% (*p* < 0.05). Sterility also showed a significant impact, contributing 9.55% (*p* < 0.05). Sterile conditions are thought to positively influence lipid content by preventing microbial contamination and reducing competition for available carbon sources, thereby supporting process stability. In contrast, Tween 80 (4.90%, *p* > 0.05) and sonication (2.68%, *p* > 0.05) exhibited relatively low contributions and were not statistically significant. Thus, while their impact on lipid content was minor compared to nitrogen and WCO concentrations, these parameters may still contribute under different cultivation strategies or in combination with other process optimizations. Furthermore, the high R^2^ (98.24%), adjusted R^2^ (93.39%), and predicted R^2^ (71.81%) values indicate that the model explains the experimental data to a large extent.

Finally, Kavšček et al. [40] reconstructed a genome-scale metabolic model of *Y. lipolytica* using baker’s yeast as a scaffold and optimized it for flux balance analysis to simulate growth and lipid production. The validated model enabled the design of a fed-batch strategy that prevented citrate excretion during lipid synthesis. Further network analysis suggested a reduced oxygen demand during lipid accumulation, and experimental reduction in aeration confirmed this prediction, resulting in an 80% increase in lipid content and more than a four-fold improvement in lipid content compared to standard conditions. Such model-guided strategies highlight the potential of integrating process optimization with systems-level analysis and may provide a valuable framework for future optimization of WCO-based SCO production processes.

**Figure 1:**
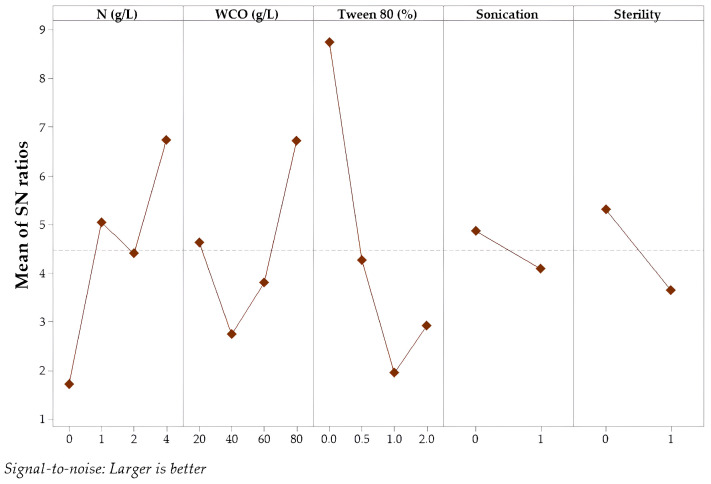
The plot of the S/N ratio for biomass production.

**Figure 2:**
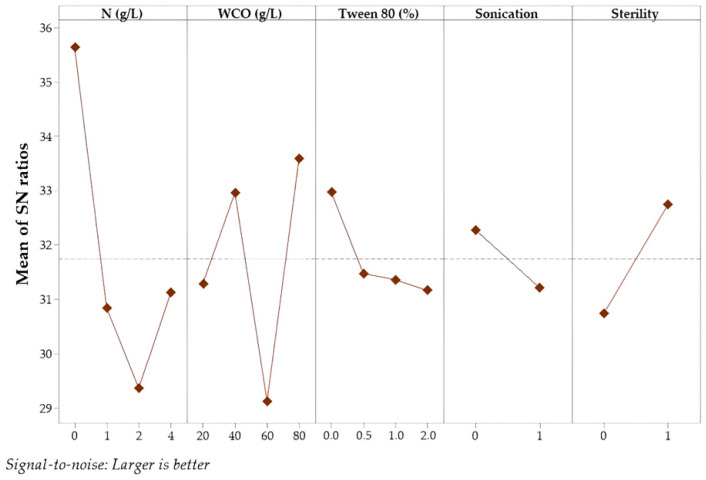
The plot of the S/N ratio for lipid content.

The main effect plots of the S/N ratio are presented in Figure 1 and Figure 2. Process optimization was conducted with the aim of enhancing the S/N ratio to improve overall process performance. The optimal level of each factor was determined as the condition that minimized experimental noise while maximizing biomass concentration and lipid content. Based on the S/N ratio analysis for biomass production, the highest mean value was observed under fermentation conditions with 4 g/L additional nitrogen, using 80 g/L WCO as the substrate, without sonication or Tween 80 addition, and under non-sterile conditions. On the other hand, the highest S/N ratio for lipid content was achieved under sterile fermentation conditions without nitrogen supplementation and Tween 80, using 80 g/L WCO as the substrate and without applying sonication.

### 3.2. Influence of Process Parameters on the Fatty Acid Composition

The fatty acid composition of lipids varied considerably under different experimental conditions (Table 5). Among the identified fatty acids, oleic acid (C18:1n9c) was the most abundant under most conditions, with the highest proportion recorded in Run 7 (56.86%), followed by Run 13 (54.74%) and Run 14 (52.45%). Although sunflower oil is typically rich in linoleic acid (C18:2n6c), the microbial lipids produced by *Y. lipolytica* were dominated by oleic acid. Notably, under nitrogen-limited conditions, exogenous fatty acids are not directly mirrored in storage lipids but are instead redistributed and complemented by de novo synthesis. This predominance of oleic acid is characteristic of the lipid profile of *Y. lipolytica* and has been consistently reported [41]. In addition, thermal oxidation during frying may reduce linoleic acid in WCO, further contributing to the observed oleic-acid predominance. In a previous study by Sayın et al. [21], fatty acid analysis of WCO revealed a clear reduction in linoleic acid content after frying.

Palmitic (C16:0) and stearic (C18:0) acids were present at higher amounts under conditions without nitrogen supplementation and low-to-moderate WCO conditions, as observed in Runs 1 and 2, where palmitic acid reached 23.11–23.80% and stearic acid up to 20.03%. In contrast, linoleic acid increased markedly under high WCO and high nitrogen conditions, with the highest proportions observed in Run 16 (40.93%) and Run 11 (38.64%). These patterns demonstrate that nitrogen and WCO concentrations significantly influence fatty acid composition. These findings align with earlier reports [42,43,44] and underscore the potential of tuning cultivation parameters to tailor fatty acid profiles for specific industrial applications such as biodiesel, nutraceuticals, and functional foods.

Taoka et al. [45] reported that the addition of Tween 80 to the culture medium significantly enhanced the growth of *Thraustochytrium aureum*, and that biomass increased proportionally with rising Tween 80 concentrations. Cultures supplemented with 1.0% Tween 80 exhibited significantly higher total lipid and fatty acid contents than the control, accompanied by a marked increase in oleic acid proportion. In another study, Katre et al. [46] evaluated several *Y. lipolytica* strains for lipid accumulation using low-cost waste-derived substrates. Among the tested feedstocks, waste cooking oil (WCO) emerged as one of the most effective carbon sources, particularly for the tropical marine strain *Y. lipolytica* NCIM 3589. This strain exhibited a notably high lipid yield coefficient of 0.43 g/g when cultivated on 100 g/L WCO, which was substantially higher than the yields reported for other waste substrates such as fish waste (0.14 g/g) and banana peel (0.09 g/g). In addition to its superior lipid productivity, the strain produced lipids enriched in saturated and monounsaturated fatty acids, predominantly oleic acid, closely resembling the fatty acid profiles of conventional vegetable oils commonly used for biodiesel production.

Taskin et al. [47] utilized deproteinized cheese whey as a substrate for lipid production by the cold-adapted yeast *Y. lipolytica* B9 under non-sterile culture conditions. Microbial contamination was successfully prevented by optimizing key culture parameters—an inoculum size of 3 mL/100 mL, initial pH of 5.5, and an incubation temperature of 15 °C. Among the tested supplements, the addition of an extra carbon source (lactose) enhanced lipid accumulation, while additional nitrogen ((NH_4_)_2_SO_4_) and phosphorus (KH_2_PO_4_) sources had no significant effect. Under optimized conditions, biomass and lipid concentrations reached 7.4 g/L and 4.29 g/L, respectively, with a lipid content of 58% of total dry biomass. The fatty acid profile comprised oleic acid, cis-10-heptadecenoic acid (C17:1), palmitoleic acid (C16:1), and palmitic acid, with no polyunsaturated fatty acids detected. The combined content of C16 and C18 fatty acids accounted for 91.98% of total lipids, while monounsaturated fatty acids represented 80.54%. Yang et al. [48] investigated the ability of *Cryptococcus curvatus* to produce microbial lipids using individual and mixed free fatty acids as substrates, aiming to design lipids with specific fatty acid compositions. When mixtures of myristic acid (C14:0), palmitic acid, and oleic acid were used, the resulting lipid compositions changed accordingly but did not fully reflect the initial substrate ratios, suggesting that oleic acid acted as a buffering fatty acid that stabilized the overall lipid profile. In mixtures containing 35% stearic acid, the proportion of this fatty acid was markedly reduced to 2.7% in the final lipid fraction, whereas the contents of palmitic acid and oleic acid increased. This observation suggests the conversion of stearic acid into palmitic acid via β-oxidation, contributing to neutral lipid biosynthesis. Overall, the produced lipids exhibited a fatty acid composition closely resembling that of palm oil. In particular, lipid fractions containing more than 60% saturated fatty acids were considered suitable as cocoa butter alternatives, whereas samples containing approximately 10% linoleic acid demonstrated potential nutraceutical value. Taskin et al. [49] investigated the lipid production potential of the yeast *Rhodotorula glutinis* TR29 in molasses-based medium under non-aseptic culture conditions. Contamination was effectively prevented using a high molasses concentration (20%) and a low initial pH (5.0). Under these optimized conditions (25 °C, 4 g/L nitrogen source, and 168 h incubation) the cell mass and lipid concentration reached 16.2 g/L and 10.5 g/L, respectively, with a lipid content of 64.8%. The major fatty acids identified in the yeast lipids were oleic (63.5%), palmitic (15.4%), stearic (9.1%), and palmitoleic acid (7.2%).

Radha et al. [50] investigated biodiesel production from SCO synthesized by *Y. lipolytica* MTCC 9520 using slaughterhouse lipid waste, specifically goat tallow, as the carbon source. Optimal cultivation conditions—96 h, pH 6, 1.5% (*v*/*v*) substrate con-centration, 5% (*v*/*v*) inoculum, and a C/N ratio of 100—yielded 3.8 g/L biomass, 2.6 g/L lipid yield, and 69.3% lipid content (g/g dry weight). Intracellular lipid accumulation was confirmed through Nile red-stained cells by fluorescence microscopy. FTIR analysis was used to characterize the extracted SCO, while gas chromatography–mass spectrometry (GC–MS) of the transesterified SCO revealed a FAME profile dominated by palmitic (42.9%), stearic (21.5%), myristic (18.3%), and oleic acids (7.0%). He et al. [51] developed an innovative lipid fermentation strategy aimed at reducing the high production costs that currently limit commercial biodiesel production. For this purpose, *Cutaneotrichosporon oleaginosum* was cultivated in a 3 L bioreactor under non-sterile conditions using bifunctional benzamide as both a selective antibacterial agent and a sole nitrogen source. When cultured on 60 g/L glucose with 1.5 g/L benzamide, the strain achieved a dry cell weight of 24.45 g/L, a lipid concentration of 15.85 g/L, and a lipid content of 64.8%, with lipid production comparable to that achieved under sterile conditions.

### 3.3. Optimization of Palmitic Acid Amount

Palmitic acid (PA) is the most abundant saturated fatty acid and plays crucial roles in various fundamental physiological processes, accounting for 20–30% of total fatty acids in the human body. It can be obtained through the diet or synthesized endogenously via de novo lipogenesis (DNL). The potential adverse health effects attributed to dietary PA are more likely driven by an imbalance in the PA/PUFA ratio and increased DNL, rather than by PA itself [52].

From an industrial perspective, palm oil represents a major commercial source, containing more than 40% PA [53]. The growing global demand for fats and oils has heightened sustainability concerns related to conventional feedstocks such as palm and soybean oil, particularly due to deforestation, intensive water consumption, and greenhouse gas emissions. In this context, SCOs produced by oleaginous yeasts have emerged as a sustainable alternative to conventional vegetable oils, as their production can utilize low-cost substrates, including agri-food wastes, thereby mitigating environmental impacts [54].

Based on the ANOVA results presented in Table 6, nitrogen concentration was identified as the most influential factor affecting PA formation, accounting for 91.16% of the total variation (F = 132.64, *p* < 0.05). Tween 80 supplementation exhibited a low yet statistically significant effect (F = 7.81, *p* < 0.05), suggesting that the surfactant may enhance lipid accessibility or facilitate fatty acid secretion. In contrast, WCO concentration, sonication, and sterilization conditions did not exert statistically significant effects (*p* > 0.05). The low residual error (0.92%) further supports the robustness and reliability of the model in describing the experimental variability. In addition, the high R^2^ (99.08%), adjusted R^2^ (96.56%), and predicted R^2^ (85.34%) values confirm the strong explanatory power of the model with respect to the experimental data.

Although Tween 80 did not exhibit a statistically significant effect on total lipid content, it played a significant role in modulating PA formation. This suggests that Tween 80 influences lipid biosynthetic pathways rather than overall lipid accumulation. Such behavior is consistent with the role of surfactants in altering membrane properties and metabolic flux distribution, thereby affecting the relative proportions of individual fatty acids [55,56,57].

**Figure 3:**
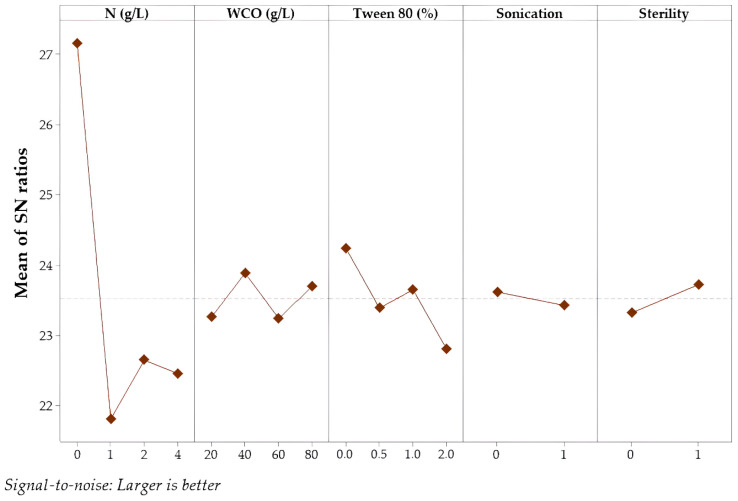
The plot of the S/N ratio for palmitic acid amount.

Figure 3 illustrates the main effect plots of the S/N ratio for PA production. As a result, the highest mean S/N ratio was achieved under fermentation conditions conducted without an additional nitrogen source or Tween 80 supplementation, using 40 g/L of WCO as the substrate, in the absence of sonication, and under sterile conditions.

## 4. Conclusions

This study demonstrated the potential of *Y. lipolytica* IFP29 for the efficient bioconversion of WCO into microbial lipids, offering a sustainable strategy within the framework of the circular bio-economy. The results confirmed that nitrogen availability and WCO concentration were the most critical parameters governing lipid accumulation, while sterilization enhanced overall process stability. Under optimized conditions, lipid content reached 72.86% of dry cell weight. In contrast, under sterilized conditions with 4 g/L additional nitrogen and 80 g/L WCO, in the absence of Tween 80 supplementation or sonication, biomass formation was favored over lipid synthesis, rendering these conditions more suitable for single-cell protein production. In addition, the third optimization results revealed that the palmitic acid (C16:0) content was significantly influenced by nitrogen availability and Tween 80 supplementation. The fatty acid profile, characterized by a high oleic acid content, further emphasized the versatility of the produced lipids for various industrial applications, including biodiesel, cosmetics, and nutraceuticals. The findings also demonstrated that culture parameters can be fine-tuned to direct fatty acid composition according to specific end-use requirements, underscoring the adaptability and flexibility of the bioprocess. Future studies should focus on scaling up the optimized process to bioreactor systems, while the application of genetic and metabolic engineering strategies may further enhance lipid productivity and substrate tolerance, thereby improving both the efficiency and economic feasibility of SCO production from WCOs.

## Figures and Tables

**Table 1:** Factors and levels for the process optimization.

Factors	Level 1	Level 2	Level 3	Level 4
Nitrogen concentration (g/L)	0	1	2	4
WCO concentration (g/L)	20	40	60	80
Tween 80 (%, *v*/*v*)	0	0.5	1	2
Sonication	0	1		
Sterility	0	1		

**Table 2:** Experimental design and S/N ratio results for biomass and lipid content.

Run	N (g/L)	WCO (g/L)	Tween 80 (%, *v*/*v*)	Sonication	Sterility	Biomass (g/L)	Lipid Content (% of Dry Cell Weight)	S/N Ratio for Biomass Production	S/N Ratio for Lipid Production
1	0	20	0.0	0	0	2.30 ± 0.03	68.56 ± 5.69	7.2346	36.7214
2	0	40	0.5	0	0	1.14 ± 0.37	58.50 ± 1.79	1.1381	35.3431
3	0	60	1.0	1	1	0.75 ± 0.03	45.96 ± 16.76	−2.4988	33.2476
4	0	80	2.0	1	1	1.12 ± 0.05	72.86 ± 4.84	0.9844	37.2498
5	1	20	0.5	1	1	1.49 ± 0.11	33.02 ± 4.81	3.4637	30.3755
6	1	40	0.0	1	1	2.16 ± 0.05	49.98 ± 0.76	6.6891	33.9759
7	1	60	2.0	0	0	1.44 ± 0.11	23.54 ± 1.65	3.1672	27.4361
8	1	80	1.0	0	0	2.20 ± 0.10	37.94 ± 3.04	6.8485	31.5819
9	2	20	1.0	0	1	1.16 ± 0.06	31.06 ± 2.27	1.2892	29.8440
10	2	40	2.0	0	1	1.12 ± 0.13	38.68 ± 4.55	0.9844	31.7497
11	2	60	0.0	1	0	2.72 ± 0.04	19.37 ± 2.90	8.6914	25.7426
12	2	80	0.5	1	0	2.15 ± 0.13	32.05 ± 1.85	6.6488	30.1166
13	4	20	2.0	1	0	2.13 ± 0.50	25.74 ± 3.03	6.5676	28.2122
14	4	40	1.0	1	0	1.28 ± 0.23	34.53 ± 0.85	2.1442	30.7639
15	4	60	0.5	0	1	1.96 ± 0.08	31.85 ± 1.60	5.8451	30.0622
16	4	80	0.0	0	1	4.18 ± 0.41	59.10 ± 1.73	12.4235	35.4317

**Table 3:** ANOVA for biomass production.

Source	DF	Seq SS	Contribution	Adj SS	Adj MS	F	*p*
Nitrogen concentration (g/L)	3	52.401	24.65%	52.401	17.4669	18.96	0.008
WCO concentration (g/L)	3	34.252	16.12%	34.252	11.4172	12.39	0.017
Tween 80 (%, *v*/*v*)	3	108.781	51.18%	108.781	36.2604	39.36	0.002
Sonication	1	2.434	1.15%	2.434	2.4338	2.64	0.179
Sterility	1	10.989	5.17%	10.989	10.9888	11.93	0.026
Error	4	3.685	1.73%	3.685	0.9213		
Total	15	212.542	100%				

DF: Degrees of freedom, Seq SS: Sequential sums of squares, Adj SS: Adjusted sum of square, Adj MS: Adjusted mean square.

**Table 4:** ANOVA for lipid content.

Source	DF	Seq SS	Contribution	Adj SS	Adj MS	F	*p*
Nitrogen concentration (g/L)	3	88.224	52.55%	88.224	29.4080	39.77	0.002
WCO concentration (g/L)	3	47.929	28.55%	47.929	15.9764	21.61	0.006
Tween 80 (%, *v*/*v*)	3	8.229	4.90%	8.229	2.7432	3.71	0.119
Sonication	1	4.501	2.68%	4.501	4.5010	6.09	0.069
Sterility	1	16.037	9.55%	16.037	16.0374	21.69	0.010
Error	4	2.958	1.76%	2.958	0.7394		
Total	15	167.879	100%				

DF: Degrees of freedom, Seq SS: Sequential sums of squares, Adj SS: Adjusted sum of square, Adj MS: Adjusted mean square.

**Table 5:** Fatty acid profile of *Y. lipolytica* under experimental conditions.

Run	N (g/L)	WCO (g/L)	Tween 80 (%, *v*/*v*)	Sonication	Sterility	C16:0	C18:0	C18:1n9c	C18:2n6c
1	0	20	0.0	0	0	23.11 ± 2.23	12.40 ± 3.54	28.76 ± 5.10	35.72 ± 0.67
2	0	40	0.5	0	0	23.80 ± 0.01	20.03 ± 0.06	45.18 ± 1.65	11.00 ± 1.72
3	0	60	1.0	1	1	21.86 ± 1.57	15.65 ± 3.83	43.64 ± 2.45	18.86 ± 4.72
4	0	80	2.0	1	1	22.54 ± 2.79	17.40 ± 5.16	44.30 ± 2.01	15.76 ± 4.38
5	1	20	0.5	1	1	12.09 ± 1.51	8.59 ± 0.01	48.35 ± 1.61	30.97 ± 3.12
6	1	40	0.0	1	1	13.94 ± 0.08	5.30 ± 0.01	42.71 ± 0.16	38.06 ± 0.06
7	1	60	2.0	0	0	10.84 ± 0.94	8.72 ± 2.26	56.86 ± 1.80	23.58 ± 1.39
8	1	80	1.0	0	0	12.62 ± 0.52	4.90 ± 0.19	47.28 ± 1.44	35.19 ± 2.15
9	2	20	1.0	0	1	14.18 ± 2.47	12.40 ± 3.68	51.14 ± 1.12	22.28 ± 7.27
10	2	40	2.0	0	1	13.14 ± 0.49	6.91 ± 0.49	46.48 ± 0.10	33.47 ± 0.88
11	2	60	0.0	1	0	14.37 ± 0.10	5.11 ± 0.89	41.88 ± 1.29	38.64 ± 2.09
12	2	80	0.5	1	0	12.66 ± 1.83	6.67 ± 1.34	46.40 ± 0.88	34.28 ± 0.40
13	4	20	2.0	1	0	11.34 ± 0.30	5.68 ± 0.74	54.74 ± 1.08	28.24 ± 0.04
14	4	40	1.0	1	0	13.73 ± 3.27	7.62 ± 0.06	52.45 ± 1.15	26.20 ± 2.18
15	4	60	0.5	0	1	13.09 ± 0.08	5.56 ± 0.91	46.51 ± 0.91	34.84 ± 1.73
16	4	80	0.0	0	1	15.24 ± 0.84	1.44 ± 0.58	42.39 ± 1.97	40.93 ± 2.23

**Table 6:** ANOVA for palmitic acid amount.

Source	DF	Seq SS	Contribution	Adj SS	Adj MS	F	*p*
Nitrogen concentration (g/L)	3	72.2554	91.16%	72.2554	24.0851	132.64	0.000
WCO concentration (g/L)	3	1.2262	1.55%	1.2262	0.4087	2.25	0.225
Tween 80 (%, *v*/*v*)	3	4.2572	5.37%	4.2572	1.4191	7.81	0.038
Sonication	1	0.1452	0.18%	0.1452	0.1452	0.80	0.422
Sterility	1	0.6489	0.82%	0.6489	0.6489	3.57	0.132
Error	4	0.7263	0.92%	0.7263	0.1816		
Total	15	79.2592	100%				

DF: Degrees of freedom, Seq SS: Sequential sums of squares, Adj SS: Adjusted sum of square, Adj MS: Adjusted mean square.

## Data Availability

The original contributions presented in this study are included in the article. Further inquiries can be directed to the corresponding author.
